# In Vitro and In Vivo Anti-Psoriasis Activity of *Ficus carica* Fruit Extracts via JAK-STAT Modulation

**DOI:** 10.3390/life13081671

**Published:** 2023-07-31

**Authors:** Jeong Hwa Lee, Mi-Young Lee

**Affiliations:** 1 Department of Medical Science, Soonchunhyang University, 22 Soonchunhyang-ro, Asan 31538, Republic of Korea; 20237438@sch.ac.kr; 2 Department of Medical Biotechnology, Soonchunhyang University, 22 Soonchunhyang-ro, Asan 31538, Republic of Korea; 3 Eshel Biopharm Co., Ltd., Soonchunhyang-ro, Asan 31538, Republic of Korea

**Keywords:** psoriasis, *Ficus carica* fruit extract, JAK–STAT modulation

## Abstract

Psoriasis, a chronic and autoimmune inflammatory disorder of the skin, has been often underdiagnosed and underestimated despite its prevalence and considerable negative effects on the quality of life. In this study, the anti-inflammatory activity of *Ficus carica* fruit extract (FFE) was investigated against LPS-stimulated RAW 264.7 cells. The in vitro results showed that FFE reduced the production of nitric oxide (NO) and iNOS expression. Moreover, FFE reduced the level of β-hexosaminidase released with histamine in allergic reactions. However, the MAPK and NFκB signaling molecules associated with the inflammatory response were not significantly regulated by FFE. In contrast, the phosphorylation of JAK1 and STAT3 in the JAK–STAT signaling pathway was dramatically reduced by FFE treatment. Psoriasis-like skin lesions were induced in BALB/c mice using imiquimod (IMQ) to test the feasibility of FFE as a treatment for psoriasis. The efficacy of FFE was evaluated based on phenotypic and histological features. FFE was effective in relieving the symptoms of psoriasis-like skin lesions, such as erythema, dryness, scales, and thick epidermis. Notably, STAT3 modulation was also contributable to the in vivo ameliorative activity of FFE. Taken together, FFE with anti-psoriasis activity in vitro and in vivo through the JAK–STAT modulation could be developed as a therapeutic agent against psoriasis.

## 1. Introduction

Psoriasis is a chronic, non-communicable, painful, disfiguring, and disabling disease that negatively affects the quality of life of patients [1]. It can occur at any age but is most common in those aged 20–30 years and 50–60 years [2]. The reported worldwide prevalence of psoriasis ranges from 0.09 to 11.4%, making it a serious global problem [3,4]. The psoriasis lesions are associated with swollen papules and silvery-white scales probably due to overactive immunity in keratinocytes provoked by IL-23 and IL-17 [5]. It has been demonstrated that Th1 overactivation can induce psoriasis, and Th17 cells play a key role in its pathogenesis and severity [6,7]. Based on the pathogenesis of this disease, psoriasis can be broadly characterized by abnormal keratinocyte division and differentiation. Symptoms, such as itching, scaling, erythema, burning, and edema, have been observed in the skin lesions of psoriasis.

In general, signal transduction pathways regulate various immune and inflammatory reactions and are involved in cell proliferation, differentiation, and apoptosis. It has been reported that an alteration in the NFκB, JAK–STAT, Akt, or Wnt signaling pathways induces psoriasis [8]. NFκB is the fundamental modulator of physiological systems, such as inflammation, development, cell cycle, proliferation, and cell death [9]. NFκB consists of the Rel family, and the major Rel/NFκB complex is the p50/p65 heterodimer [10]. Inactive +NFκB dimers are located in the cytoplasm and bind with the IκB family of inhibitory proteins, including IκBα [11]. The phosphorylation of IκBα by the IKK complex triggers liberation of the NFκB dimer, leading to the entering of NFκB into the nucleus and triggering the inflammation cascade. Psoriasis, as an inflammatory skin disorder, is marked by elevated and activated NFκB via phosphorylation [12].

The mammalian family of mitogen-activated protein kinases (MAPKs) include extracellular signal-regulated kinase (ERK), p38, and c-Jun NH2-terminal kinase (JNK) [12]. The MAPK pathways are activated by diverse extracellular and intracellular stimuli, including cytokines, hormones, and various cellular stressors that include oxidative stress and endoplasmic reticulum stress [13]. The potential role of the p38 MAPK pathway has been suggested to be involved in the inflammatory pathogenesis of psoriasis [14].

Members of the mammalian Janus kinase (JAK) and signal transducer and activator of transcription (STAT) families have been extensively analyzed in mouse and human systems [15]. Four JAKs—JAK1, JAK2, JAK3, and Tyk2—and seven STATs—STAT1, STAT2, STAT3, STAT4, STAT5A, STAT5B, and STAT6—were identified [15]. Upon phosphorylation of JAK, STAT and other cytokines are subsequently phosphorylated. Drugs that inhibit the JAK–STAT signaling pathway are known as JAK inhibitors, including tofacitinib, ruxotinib, and baricitinib [8]. Psoriasis is associated with the overexpression of JAK1 and STAT3 [8]. Thus, the JAK and STAT inhibitors can be used as therapeutics to treat psoriasis [16].

*Ficus carica* is used in traditional medicine for a wide range of ailments related to digestive, endocrine, reproductive, respiratory, gastrointestinal, and urinary tract infections [17,18]. *Ficus carica* contains a variety of coumarins and flavonoids in its fruits and leaves, and these compounds are known to have antioxidant, anti-cancer, and anti-inflammatory effects [19,20]. Psoralen is one of the components of *F. carica* that has been used as a drug for skin disease [21]. Psoralen is currently an FDA-approved treatment under the name PUVA therapy [22] and is an effective treatment for ailments, such as eczema, psoriasis, and vitiligo, when co-treated with UV-A. As a photosensitizer, psoralen must be exposed to UV-A radiation for therapeutic efficacy.

The leaves and fruits of *F. carica* differ not only in chemical composition but also in efficacy and toxicity [17]. Studies on the efficacy of *F. carica* fruit extract on inflammation in psoriasis have not been available until now. In this study, the in vitro ameliorative effects of *F. carica* fruit extract were analyzed in LPS-activated RAW 264.7 cells. The in vivo suppressing effect of the extract on the inflammatory response was also investigated using a psoriasis-like mouse model.

## 2. Materials and Methods

### 2.1. In Vitro Study

#### 2.1.1. Plant Extracts

The methanolic extracts of *F. carica* fruit used in this study were obtained from the Korea Plant Extract Bank at the Korea Research Institute of Bioscience and Biotechnology (Daejeon, Republic of Korea). The fruit (31 g), which was dried in the shade and powdered, was added to 1 L of methyl alcohol 99.9% (HPLC grade) and extracted through 30 cycles (40 KHz, 1500 W, 15 min ultrasonication and 120 min standing per cycle) at room temperature using an ultrasonic extractor (SDN-900H, SD-ULTRASONIC CO., LT., Seoul, Republic of Korea). After filtration (Qualitative Filter No. 100, HYUNDAI MICRO CO., LT., Seoul, Republic of Korea) and drying under reduced pressure, *F. carica* extract (8.4 g) was obtained and diluted to a 5% stock solution using dimethyl sulfoxide (DMSO).

#### 2.1.2. DPPH Free Radical Scavenging Assay

Diluted *Ficus carica* fruit extract (FFE) at various concentrations was added to the DPPH ethanolic solution, and the volume of the reaction mixture was adjusted to 200 μL. Each mixture was suspended vigorously and incubated in the dark at 37 °C for 30 min. The absorbance was measured at 517 nm. The DPPH free radical scavenging activity was calculated according to this formula:

$$Scavenging\;activity\left(\%\right)=\frac{1-\left({Abs}_{FFE}-{Abs}_{blank}\right)}{A{bs}_{control}}\times 100$$

where Abs_FFE_ is the absorbance of the FFE, Abs_blank_ is the absorbance of the blank, and Abs_control_ is the absorbance of the control. Ascorbic acid was used as the positive control.

#### 2.1.3. Determination of Total Polyphenol Content

Total polyphenol content was determined using the Folin–Ciocalteu assay with some modifications [23]. Briefly, 750 μL of the samples and gallic acid were mixed with 150 μL Folin–Ciocalteu’s phenol reagent and incubated for 5 min at room temperature. A total of 150 μL of 20% Na_2_CO_3_ was then added and incubated for 30 min at 40 °C. After incubation, the absorbance of the reaction mixture was measured at 750 nm. A gallic acid curve was used to quantify the total polyphenol content. The results were expressed as milligrams (mg) of gallic acid equivalents (GAE). The total phenolic contents in the FFE were calculated using this formula:

C=cV/m

where C = total phenolic content mg GAE/g of FFE, c = concentration of gallic acid obtained from the calibration curve in mg/mL, V = volume of the extract in mL, and m = mass of the extract in grams.

#### 2.1.4. Determination of Total Flavonoid Content

Total flavonoid content was determined using the aluminum chloride (AlCl_3_) colorimetric method with some modifications [24]. The entire process was performed at room temperature. Briefly, 400 μL of the samples and quercetin were mixed with 30 μL of 5% NaNO_2_ for 5 min. Thirty microliters of 10% AlCl_3_ was added, and the reaction mixture was incubated for 5 min. A total of 400 μL of 4% NaOH was added and incubated at room temperature for 15 min. Finally, the mixture was adjusted with distilled water to a final volume of 1 mL and gently mixed. The absorbance was measured at 510 nm. A quercetin curve was used to quantify the total flavonoid content. The results were expressed as milligrams (mg) of quercetin equivalents (QE). The total flavonoid contents in the FFE were calculated using this formula:

C=cV/m

where C = total flavonoid content mg QE/g of FFE, c = concentration of quercetin obtained from the calibration curve in mg/mL, V = volume of the extract in mL, and m = mass of the extract in grams.

#### 2.1.5. LC–MS/MS

Chemical components were analyzed using a liquid chromatography–tandem mass spectrometry (LC–MS/MS) system, specifically employing the Thermo TSQ Altis system coupled to a Thermo 3000 RSLC with a quaternary gradient pump and autosampler (Thermo Fisher Scientific, Waltham, MA, USA) integrated with the Vanquish Flex UHPLC system. Chromatographic separation was carried out on an Agilent C18 column. The mobile phase was comprised of distilled water (DW) and acetonitrile (ACN), both with added acetic acid. The column temperature and sample organizer were maintained at 40 °C and 15 °C, respectively. Elution from the column was accomplished using a gradient method at a constant flow rate of 0.3 mL/min with the following conditions: from 5% to 95% of solvent B over 2 min, a 2 min wash with 100% B, and a 2 min re-equilibration with 5% B. The injected sample volume was 1.0 μL. Analyses were performed in the optimized data-dependent acquisition (DDA) mode via negative ion mode electrospray ionization (ESI-). The analyses constituted a full MS survey scan within the *m*/*z* range of 100–2000 Da (scan time: 150 ms) and MS/MS scans for the three most intense ions. Collision energy was applied in a stepped manner from 30 V to 100 V. ESI parameters were set as follows: capillary voltage at 2.9 kV, cone voltage at 40 V, source temperature at 120 °C, desolvation temperature at 350 °C, cone gas flow at 50 L/h, and desolvation gas flow at 800 L/h. High-purity nitrogen was used as the nebulizer and auxiliary gas, while argon served as the collision gas.

#### 2.1.6. Nitric Oxide Assay

RAW 264.7 cells, a murine macrophage cell line, were seeded 1 × 10^6^ cells/well on a 6-well plate and incubated for 24 h. FFE was added into the wells with the LPS (1 μg/mL) and incubated for 18 h at 37 °C in a CO_2_ incubator. The amounts of nitrite formed were measured using Griess reagent (0.2% naphthylethylenediamine dihydrochloride and 2% sulfanilamide in 10% H_3_PO_4_). Briefly, 100 μL of cell supernatant was mixed with 150 μL of Griess reagent. The absorbance was measured at 540 nm using a microplate reader (Biotek, Synergy HTX, Winooski, VT, USA). The concentration of nitrite was determined from a sodium nitrite standard curve.

#### 2.1.7. Western Blot

The cells were lysed by adding RIPA buffer (50 mM Tris-HCl, pH 7.4, 150 mM NaCl, 1% Triton X-100, 0.5% sodium deoxycholate, 0.1% SDS, and 2 mM EDTA) with 1% (*v*/*v*) protease inhibitor cocktail (PIC) and 1% (*v*/*v*) phenylmethylsulfonyl fluoride (PMSF) and then sonicated twice for 20 s at 10 s intervals. Then, the cells were centrifuged at 15,000 rpm at 4 °C for 50 min. The supernatant was collected for the Bradford protein quantification assay [25]. Equal amounts of protein were loaded onto a 10% sodium dodecyl sulfate-polyacrylamide 10% SDS-PAGE gel and electrophoretically separated for 1 h. The proteins were then transferred to gels and nitrocellulose (NC) membranes for 1 h at 400 mA. The transferred NC membranes were blocked in 5% skim milk in TBST buffer (Tris-buffered saline, 0.1%) at 4 °C overnight. The membranes were then incubated for 2 h at room temperature with individual primary antibodies and washed three times with TBST buffer. Then, the membranes were incubated for 1 h at room temperature with secondary antibodies and developed using ECL reagents. The relative intensities of the Western blot bands were quantified using β-actin.

#### 2.1.8. β-Hexosaminidase Assay

RBL-2H3 cells, a murine basophil cell line, were seeded 2 × 10^5^ cells/well on a 6-well plate and incubated for 24 h. The cells were then treated with 2,4-dinitrophenyl (DNP)-IgE (100 ng/mL) overnight. To remove the excess DNP-IgE, the cells were washed twice with the Siraganian buffer (119 mM NaCl, 5 mM KCl, 5.6 mM glucose, 25 mM PIPES, 0.4 mM MgCl_2_, 1 mM CaCl_2_, 0.1% BSA, and pH 7.2). FFE diluted in the Siraganian buffer was added to the cells and incubated for 1 h. The cells were stimulated with DNP-BSA (100 ng/mL) for 2 h. After incubation, the supernatant was collected and incubated with 2 mM p-nitrophenyl-N-acetyl-β-d-glucosaminide (p-NAG) in 0.1 M citrate buffer (pH 4.5). The enzyme reaction was terminated by adding 0.1 M sodium carbonate buffer (pH 10), and the absorbance was measured at 405 nm.

### 2.2. In Vivo Study

#### 2.2.1. Animals

Six-week-old male BALB/c mice were purchased from ORIENT Inc. (Sungnam, Republic of Korea). The mice were kept in cages and maintained at 25 °C under a 12 h light/12 h dark cycle during the entire experiment. The animal experiments were approved by the Institutional Animal Care and Use Committee (SCH22-0053).

#### 2.2.2. Psoriasis-like Mouse Model

The dorsal hair of each group of five mice was removed with hair removal cream. The mice were divided into six groups (*n* = 5): normal (non-treated group), IMQ (negative control group), dexamethasone (DEX, positive control group, 5 mg/mL dexamethasone in autoclaved DDW), FH (high-dose *Ficus carica* fruit extract, 10 mg/mL), FM (moderate-dose *Ficus carica* fruit extract, 5 mg/mL), and FL (low-dose *Ficus carica* fruit extract, 1 mg/mL). Inflammation was induced by local administration of 62.5 mg of IMQ to the dorsal skin and ear once every 7 d. Eight days after the first administration, all groups were sacrificed, and the dorsal skin, ear, and spleen were collected.

#### 2.2.3. Western Blot

Mouse dorsal skin was lysed by adding RIPA buffer (50 mM Tris-HCl, pH 7.4, 150 mM NaCl, 1% Triton X-100, 0.5% sodium deoxycholate, 0.1% SDS, and 2 mM EDTA) with 1% (*v*/*v*) protease inhibitor cocktail (PIC) and 1% (*v*/*v*) phenylmethylsulfonyl fluoride (PMSF) and then sonicated four times for 20 s at 5 s intervals. Then, the lysate was centrifuged at 15,000 rpm at 4 °C for 50 min. The subsequent processes were the same as those used in Section 2.1.7.

#### 2.2.4. Histological Analysis

The dorsal skin was collected and fixed with 10% formalin. After fixation, the skin was dehydrated and embedded in paraffin. The tissues were cut into 3 μm sections. Hematoxylin and eosin (H&E) staining was performed to measure epidermal thickness.

### 2.3. Statistical Analysis

All results are expressed as the mean ± SD and evaluated by one way ANOVA to confirm the significance between groups. Comparisons of three or more groups were performed using Scheffe’s post-hoc test. Data were considered significantly different at *p* < 0.05.

## 3. Results

### 3.1. Antiradical Activity and Chemical Constituents

Table 1 shows the antiradical activity of *Ficus carica* fruit extracts determined using the DPPH assay. The total polyphenol and flavonoid contents in FFE are also indicated. The value at which the concentration scavenges 50% of free radicals (IC_50_) was 626.52 ± 24.75 μg/mL, showing significant radical scavenging activity. The total polyphenol and flavonoid contents were 56.94 ± 0.01 mg GAE/g and 14.71 ± 0.25 mg QE/g, respectively. The results showed that the antiradical activity may arise from the presence of polyphenols and flavonoids. The phytochemicals in the extract were directly analyzed by LC–MS/MS. The sixteen identified compounds are listed in Table 2.

### 3.2. In Vitro Anti-Inflammatory Effect of Ficus carica Fruit Extract in LPS-Stimulated RAW 264.7 Cells

**Figure 1:**
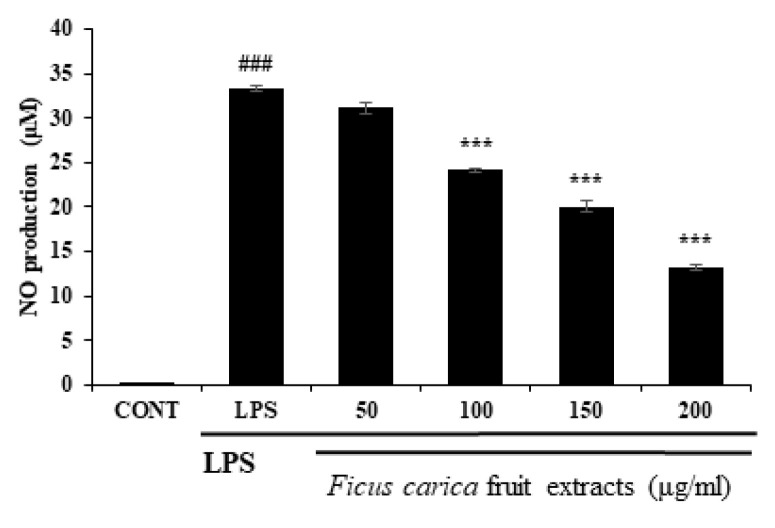
Effect of *F. carica* fruit extracts on nitric oxide production of RAW 264.7 cells. *Ficus carica* fruit extracts significantly inhibit NO production of LPS-stimulated RAW 264.7 cells in a dose-dependent manner. All data are expressed as mean ± SD *** *p* < 0.001 compared with control group, ^###^ *p* < 0.001 compared with LPS group. All experiments were performed in triplicate.

**Figure 2:**
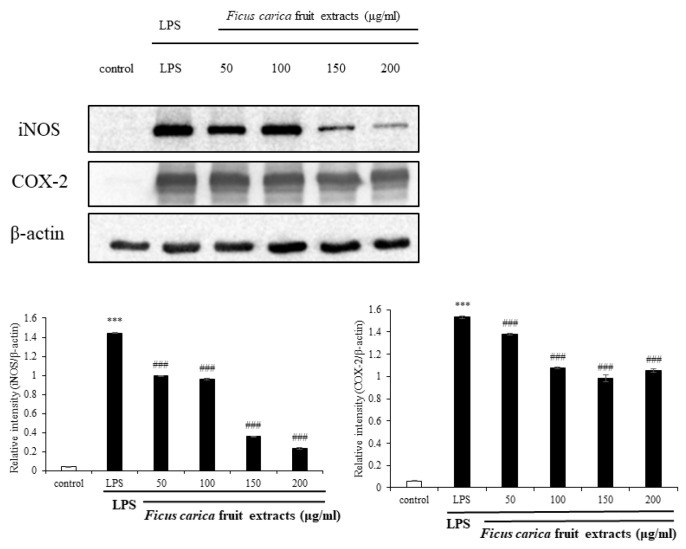
Effect of *F. carica* fruit extracts on iNOS and COX-2 expressions of RAW 264.7 cells. Analysis of the expression levels of iNOS and COX-2 were performed with ImageJ and normalized against β-actin. All data are expressed as mean ± SD *** *p* < 0.001 compared with control group, ^###^ *p* < 0.001 compared with LPS group. All experiments were performed in triplicate.

To investigate the in vitro anti-inflammatory efficacy of FFE, FFE dissolved in DMSO at the non-cytotoxic dose was applied to the LPS-stimulated RAW 264.7 cells. As a result of the NO assay using Griess reagent through the cell supernatant, FFE inhibited NO production in the RAW 264.7 cells in a concentration-dependent manner (Figure 1). During the inflammatory reaction, NFκB enters the nucleus, and various inflammatory molecules, including iNOS and COX-2, are produced. iNOS catalyzes the formation of NO from L-arginine, leading to NO production in response to the inflammatory reaction [41]. As shown in Figure 2, Western blot was performed to investigate the expression of the inflammatory marker proteins in the RAW 264.7 cells. LPS-induced inflammatory marker proteins, such as iNOS and COX-2, were reduced by treatment with FFE (Figure 2).

### 3.3. Effect of F. carica Fruit Extract on the MAPK and NFκB Signaling Pathways in RAW 264.7 Cells

**Figure 3:**
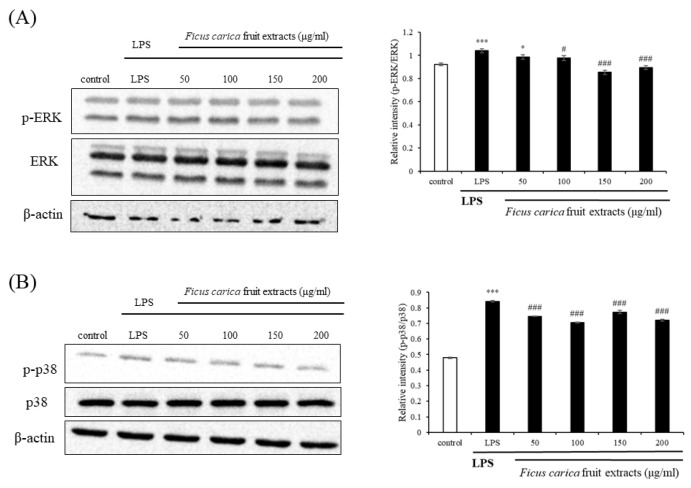
Effect of *F. carica* fruit extracts on the phosphorylation of mitogen-activated protein kinase (MAPK) cascade (p-ERK 1/2 and p-p38) in LPS-stimulated RAW 264.7 cells. The expressions of p-ERK 1/2 (**A**) and p-p38 (**B**) were analyzed with ImageJ and normalized against β-actin. All data are expressed as mean ± SD * *p* < 0.05, *** *p* < 0.001 compared with control group, ^#^ *p* < 0.05, ^###^ *p* < 0.001 compared with LPS group. All experiments were performed in triplicate.

Western blotting of proteins belonging to the inflammatory pathways was performed to examine the underlying mechanism that relieves inflammation. The MAPK signaling pathways include ERK, JNK, and p38 [42]. ERK and p38 phosphorylation was slightly reduced by FFE treatment (Figure 3B). This result suggests that the anti-inflammatory efficacy of FFE may not be closely related to the MAPK signaling pathway.

**Figure 4:**
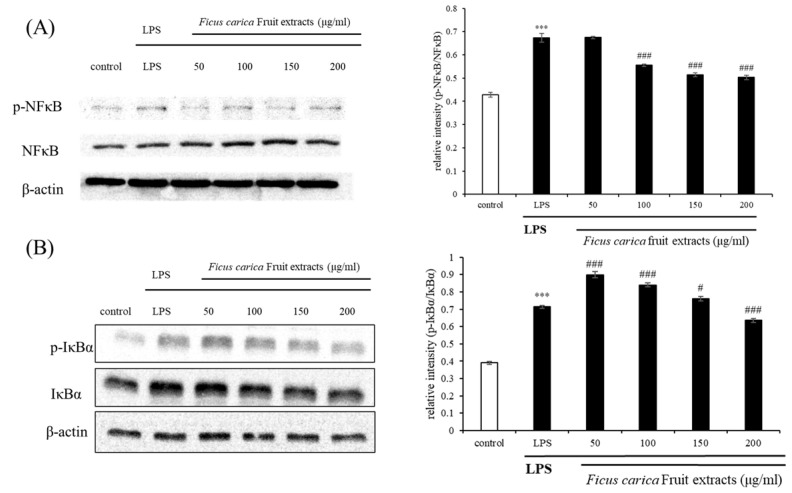
Effect of *F. carica* fruit extract on the LPS-stimulated activation of phosphorylation of NFκB (**A**) and IκBα (**B**) in RAW 264.7 cells. The expression of p-NFκB and p-IκBα was analyzed with ImageJ and normalized against β-actin. All data are expressed as mean ± SD *** *p* < 0.001 compared with control group, ^#^ *p* < 0.05, ^###^ *p* < 0.001 compared with LPS group. All experiments were performed in triplicate.

In the physiologically normal state (absence of inflammation), cytoplasmic IκBα prevents NFκB from entering the nucleus. However, once IκBα is phosphorylated due to inflammation, it cannot properly function as an NFκB inhibitor, allowing NFκB to freely enter the nucleus. Then, the NFκB that enters the nucleus acts as a transcriptional regulator, releasing various inflammatory cytokines and chemokines [43]. Figure 4 shows that FFE inhibits the phosphorylation of NFκB and IκBα to some extent.

### 3.4. Regulatory Effect of Ficus carica Fruit Extracts on the JAK–STAT Signaling Pathway

**Figure 5:**
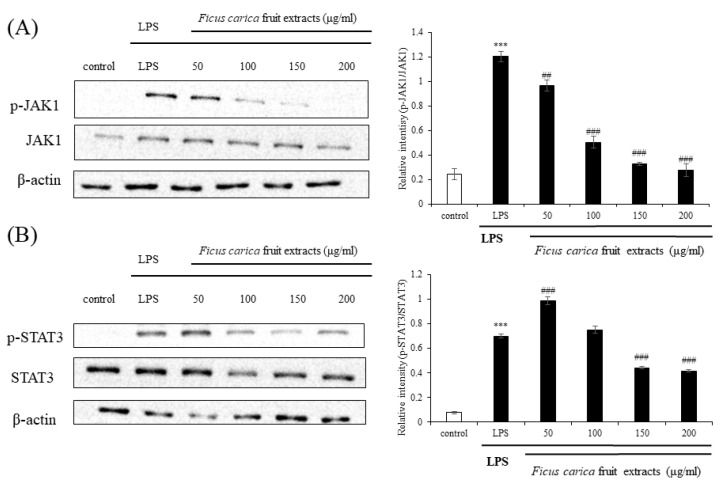
Effect on *F. carica* fruit extracts on p-JAK1 and p-STAT3 expressions of RAW 264.7 cells. The expressions of p-JAK1 (**A**) and p-STAT3 (**B**) were analyzed with ImageJ and normalized against β-actin. All data are expressed as mean ± SD *** *p* < 0.001 compared with control group, ^##^ *p* < 0.01 compared with LPS group, ^###^ *p* < 0.001 compared with LPS group. All experiments were performed in triplicate.

Currently, the JAK–STAT signaling pathway is emerging as a new inflammatory pathway following the classical and traditional MAPK and NFκB signaling. When cytokines, such as IL-6, IL-19, and IL-22, bind to their receptors, JAK is phosphorylated and binds to the cytokine receptor to make a receptor complex, resulting in the phosphorylation of downstream STAT3 at Tyr705 and Ser727, and the phospho-STAT3 meet to form dimers. These dimers bind to the nuclear DNA and regulate the expression of inflammatory proteins [44,45]. Western blotting showed that the expression levels of LPS-induced p-JAK1 and p-STAT3 were drastically reduced following FFE application (Figure 5). These results suggest that FFE sufficiently inhibits inflammation in vitro by acting as an inhibitor of the JAK–STAT pathway rather than as the classically accepted inhibitor of the MAPK and NFκB pathways.

### 3.5. Effect of Ficus carica Fruit Extracts on the β-Hexosaminidase Release

**Figure 6:**
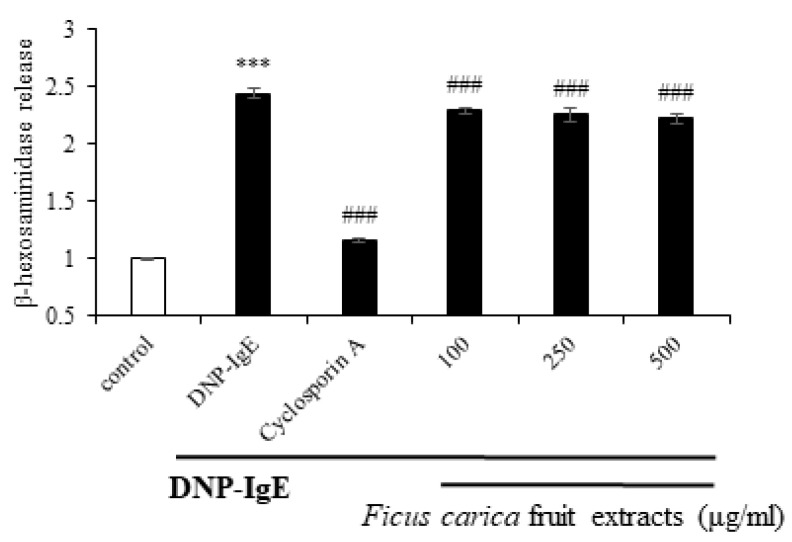
Effect of *F. carica* fruit extracts on β-hexosaminidase release in DNP-IgE-stimulated RBL-2H3 cells. The data are the mean ± SD. *** *p* < 0.001 compared with control group, ^###^ *p* < 0.001 compared with DNP-IgE. All experiments were performed in triplicate.

The β-hexosaminidase assay was performed to investigate the anti-allergic activity of FFE. β-hexosaminidase is an enzyme that is released together with histamine and is involved in allergic inflammation. β-hexosaminidase could be used as a biomarker for an allergic reaction since it is released with histamine [46]. To investigate the release of β-hexosaminidase at the cellular level, we used the rat basophilic leukemia cell line, RBL-2H3. The cells were sensitized using DNP-IgE, and DNP-BSA was used as an antigen to induce allergic reactions. The amount of secreted β-hexosaminidase bound to the substrate was determined. Figure 6 shows that the negative control group, treated with only DNP-IgE, elevated β-hexosaminidase release by approximately 2.4 times compared to the control group. However, upon treatment with FFE, the release of β-hexosaminidase was downregulated compared to that in the negative control group. These data suggest that β-hexosaminidase release is inhibited by FFE, resulting in a reduction in inflammation.

### 3.6. IMQ-Induced Psoriasis in BALB/c Mice

**Figure 7:**
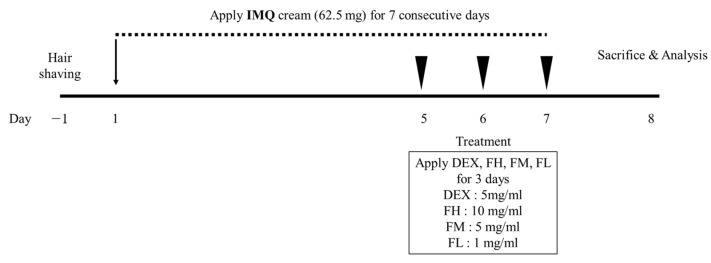
The scheme of animal experiments conducted to investigate the psoriasis-improvement effect of *F. carica* fruit extracts. IMQ, Imiquimod; DEX, dexamethasone; FH, high-dose *Ficus carica* extract; FM, moderate-dose *Ficus carica* extract; FL, low-dose *Ficus carica* extract.

**Figure 8:**
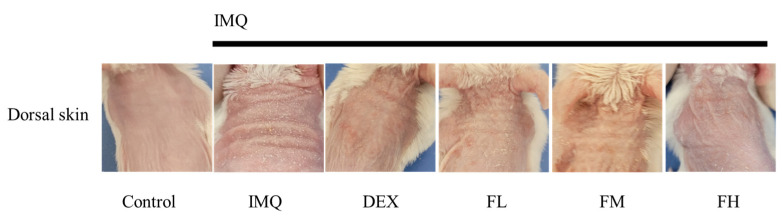
Phenotypical observations of dorsal skin in mice with IMQ-induced psoriasis-like skin lesions. Control mice with daily topical application of Vaseline on the shaved dorsal skin. Test mice with daily IMQ-treated (62.5 mg) dorsal skin on day 3 after IMQ treatment showing psoriasis-like inflammation and erythema lesions and silvery-white scales. IMQ, imiquimod; DEX, dexamethasone; FH, high-dose *Ficus carica* extract, FM, moderate-dose *Ficus carica* extract; FL, low-dose *Ficus carica* extract. All experiments were performed in triplicate.

Figure 7 shows the scheme of the animal experiments conducted to investigate the psoriasis-ameliorating effect of FFE. Psoriatic skin lesions were excessively induced by daily treatment with 62.5 mg of IMQ on the dorsal skin of mice for 7 days. At 5, 6, and 7 d, IMQ cream was applied in the morning to develop and maintain psoriasis, and then FFE was applied in the afternoon. FFE was divided into three doses: high, moderate, and low. Figure 8 shows the phenotypical results of the animal experiments. Simple phenotypic features, such as dryness, scales, and erythema, were excessively induced in the psoriasis-induced group using only IMQ. However, symptoms including scales and erythema were alleviated in a concentration-dependent manner in the FFE group, and an almost superior improvement was observed in the high dose (FH) group similar to that in the dexamethasone-treated group.

### 3.7. Measurement of PASI Score in BALB/c Mice

**Figure 9:**
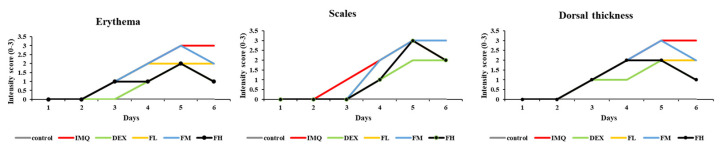
PASI scores showing intensity of erythema and scales of the control and treated mice dorsal skin on a 0–3 point scale. All experiments were performed in triplicate.

The PASI score evaluates the severity of psoriasis by measuring the degree of redness (erythema), thickness, scales of the psoriasis lesion, and the extent of psoriasis spread. Figure 9 shows that all indicators, such as erythema, scales, and dorsal thickness, steadily increased in the IMQ-treated group. However, the scores for erythema, scales, and dorsal skin thickness decreased after FFE treatment. In particular, the high-concentration FFE treatment group showed an improvement similar to that of the positive control group (dexamethasone).

### 3.8. Histological Analysis of Effect of F. carica Fruit Extracts on Psoriasis-like Skin Lesions in BALB/c Mice

**Figure 10:**
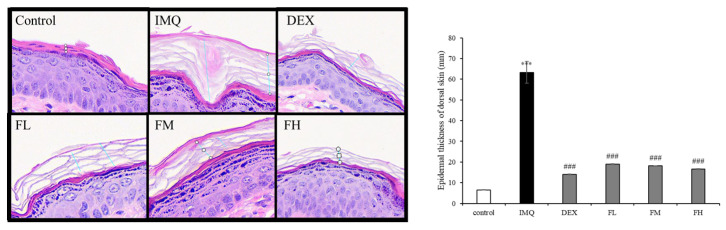
Histological examinations stained with hematoxylin and eosin (H&E). H&E-stained dorsal skin of control and IMQ-treated mice. Images magnified 40×. *** *p* < 0.001 compared with control group, ^###^ *p* < 0.001 compared with IMQ. All experiments were performed in triplicate.

Figure 10 shows that the thickness of the stratum corneum increased in the IMQ-treated group, which caused psoriasis. However, FFE treatment reduced the epidermal thickness, and the high-dose FFE treatment reduced the level of scales comparable to that of dexamethasone.

### 3.9. Effect of Ficus carica Fruit Extracts on the Spleen Weight

**Figure 11:**
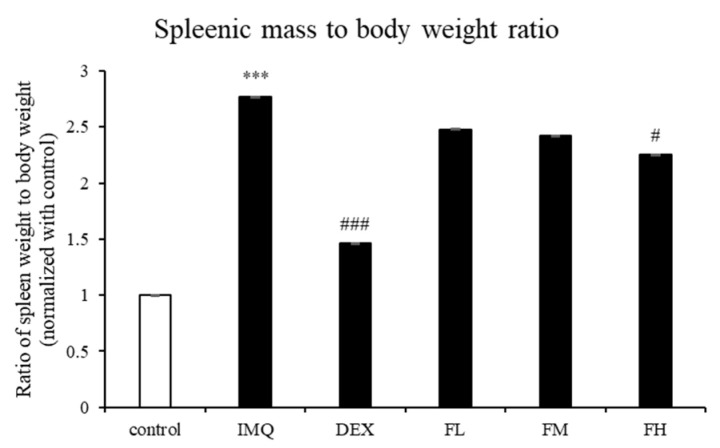
Effect of *F. carica* fruit extracts on the ratio of spleen weight to body weight. Mice were sacrificed, and the ratio of spleen weight to body weight was determined at 24 h after the final administration. Data presented are mean ± SD (*n* = 5) *** *p* < 0.001 indicates a statistically significant difference from the control group. ^#^ *p* < 0.05, ^###^ *p* < 0.001 indicates a statistically significant difference from the IMQ group. All experiments were performed in triplicate.

The spleen is a representative immune organ, and an enlarged spleen indicates excessive inflammation in the body [47]. The size of the spleen significantly increased when only IMQ was applied. However, the weight of the spleen was significantly reduced in the high-dose FFE group compared to that in the IMQ group (Figure 11).

### 3.10. Effect of F. carica Fruit Extracts on the Phosphorylation of STAT3 in Mouse Dorsal Skin

**Figure 12:**
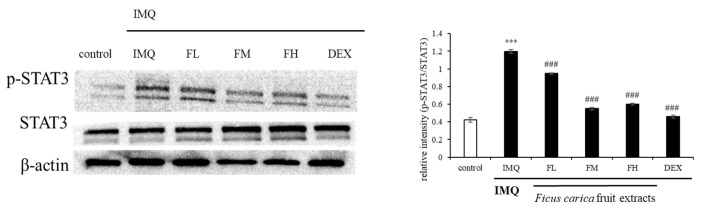
Effect of *F. carica* fruit extracts on the phosphorylation of STAT3. The expression of p-STAT3 was analyzed with ImageJ and normalized against β-actin. All data are expressed as mean ± SD *** *p* < 0.001 compared with control group, ^###^ *p* < 0.001 compared with IMQ group. All experiments were performed in triplicate.

To investigate the mechanism underlying the in vivo ameliorative effect of FFE on psoriasis-like skin lesions, the p-STAT3 expression pattern was investigated. Figure 12 shows that p-STAT3 was overexpressed in the group treated with IMQ alone. However, the IMQ-induced p-STAT3 expression was reduced by FFE treatment. These results indicated that FFE improved the symptoms of psoriatic skin lesions by regulating STAT3 expression.

## 4. Discussion

Psoriasis is an immune-mediated, long-lasting inflammatory skin disease [48] characterized by great risk of morbidity, chronicity, disability, and associated comorbidities [49]. The common occurrence of psoriasis on the skin primarily involves an inflammatory response involving IFN-α, IFN-γ, IL-1, IL-6, IL-17, IL-22, and IL-23 from dendritic cells, macrophages, and helper T cells (Th cells) [50,51]. Activation of NF-κB-mediated inflammatory events involving iNOS and COX2 is known to be associated with psoriasis initiation and progression. The pathogenesis of psoriasis is complex, and the contribution of both innate and adaptive immunity might help manage this complex disease [52,53].

An extensive literature survey revealed that *F. carica* is a sacred and important medicinal plant used for the ethnomedicinal treatment of anemia, bronchitis, constipation, diabetes, fever, hemorrhoids, inflammation, liver disorders, infectious diseases, and many other ailments worldwide [7,17]. Pharmacological studies on fresh plant materials, crude extracts, and isolated components of *F. carica* have provided evaluations of their anti-bacterial, anti-cancer, anti-fungal, anthelmintic, anti-inflammatory, anti-mutagenic, anti-pyretic, anti-spasmodic, anti-platelet, antiviral, cytotoxic, hepatoprotective, hypoglycemic, hypolipidemic, and immunostimulant activities [15].

In this study, the in vitro and in vivo anti-psoriasis effect of *F. carica* fruit extract (FFE) was investigated. The in vitro results showed that FFE reduced the production of nitric oxide (NO), iNOS, and COX-2 from LPS-stimulated RAW 264.7 cells. This anti-inflammatory activity is thought to be due to the basic antioxidant activity of the extract, probably owing to the presence of polyphenols and flavonoids.

The MAPK and NFκB signals, which are typical inflammatory pathways, were not significantly regulated by FFE. Currently, the JAK–STAT signaling pathway is emerging as the main mechanism underlying psoriasis pathogenesis. Interestingly, phosphorylation of JAK1 and STAT3 in the x JAK–STAT signaling pathway, which has been highlighted as a new inflammatory signal, was dramatically modulated by FFE. In addition, FFE reduced the release of β- hexosaminidase in the anti-allergic experiments.

When psoriasis was induced in BALB/c mice with IMQ, the dorsal thickness of the mice increased. However, this increased dorsal thickness was significantly reduced by FFE treatment. Moreover, the PASI score was reduced with FFE. In addition, histological observations revealed that the dermal thickness of the epidermis was decreased. The size and weight of the spleen were also reduced. Notably, FFE reduced the phosphorylation of STAT3 in the psoriasis-like mouse model.

Taken together, *F. carica* fruit extract might show a superior ameliorative effect on psoriatic skin lesions via anti-inflammatory effects associated with JAK–STAT modulation. Thus, the extract could be developed as a candidate material for the treatment of psoriasis. In further experiments, *F. carica* fruit extract will be fractionated to find the ingredient with the highest anti-psoriasis efficacy and developed as a drug with high specificity.

## Figures and Tables

**Table 1:** Antiradical activities of *F. carica* fruit extracts.

Plant Parts	Antiradical Activity	Total Phenolics (mg GAE/g)	Total Flavonoids (mg QE/g)
	DPPH IC_50_ (μg/mL)		
Fruit	626.52 ± 24.75	56.94 ± 0.01	14.71 ± 0.25

Values are given as mean ± SD of the triplicate experiments.

**Table 2:** Chemical constituents of *F. carica* fruit extracts by LC–MS/MS.

Compound	Concentration (mg/kg)	Efficacy [Ref.]
Rutin	2254.58	Anti-radical [26]
Chlorogenic acid	1974.34	Anti-radical [27]
Protocatechuic acid	1244.37	Anti-radical [28]
Psoralen	1037.41	Anti-psoriasis [29]
Schaftoside	860.55	Anti-inflammation [30]
Orientin	400.83	Anti-radical [31]
Bergapten	355.51	Anti-inflammation [32]
Caffeoylmalic acid	326.92	N.D
Vitexin	278.8	Anti-inflammation [33]
Fraxin	190.38	Anti-IR injury [34]
Cichoriin	137.55	Anti-radical [35]
Sinapic acid	119.06	Anti-inflammation [36]
Loganic acid	93.73	Anti-radical [37]
Sweroside	56.24	Anti-inflammation [38]
Salidroside	51.51	Anti-inflammation [39]
Nodakenetin	24.85	Anti-inflammation [40]

N.D (Not determined).

## Data Availability

Data is unavailable.
